# Case Report: Two cases of adrenal hemangioma and literature analysis

**DOI:** 10.3389/fonc.2025.1588361

**Published:** 2025-09-01

**Authors:** Xiaochen Dong, Min Wang

**Affiliations:** ^1^ Clinical Medical College, Jining Medical University, Jining, Shandong, China; ^2^ Department of Radiology, Jining No.1 People’s Hospital Affiliated to Shandong First Medical University, Jining, Shandong, China

**Keywords:** adrenal gland, incidentaloma, angioma, magnetic resonance imaging, x-ray computed tomograghy

## Abstract

Adrenal hemangioma is a rare benign tumor that is often misdiagnosed due to its low incidence and limited clinical awareness. We report two cases of adrenal hemangioma and provide a detailed description of their CT and MRI imaging features, surgical findings, and pathological characteristics. Both patients were elderly males, and the adrenal hemangiomas were incidentally discovered during imaging examinations. The lesions were unilateral, with one case on the left side and the other on the right side. The sizes were approximately 3.4 cm × 3.6 cm × 3.5 cm and 6.7 cm × 9.0 cm × 8.8 cm, respectively. The margins were well-defined, and both lesions were quasi-circular. On Fat-suppressed T2-weighted (T2WI-FS), the lesion showed heterogeneous hyperintensity, while on Diffusion-weighted imaging (DWI), the lesion demonstrated no restricted diffusion. On contrast-enhanced CT and MR imaging, the lesion shows peripheral nodular enhancement with delayed centripetal filling. Additionally, a well-defined, rim-enhancing tumor capsule is observed. Our case report aims to increase awareness of this rare adrenal tumor.

## 1 Introduction

Hemangioma is a benign, non-functional vascular tumor that rarely occurs in the adrenal gland, accounting for only 0.01% of adrenal tumors (1). Adrenal hemangiomas typically do not have endocrine function, and patients are usually asymptomatic. When the lesion compresses surrounding structures, it may cause abdominal pain, and rupture of the lesion can lead to retroperitoneal hemorrhage (2). Preoperative imaging diagnosis of adrenal hemangiomas is rarely accurate, and blind biopsy carries a significant risk of hemorrhage.

**Figure 1:**
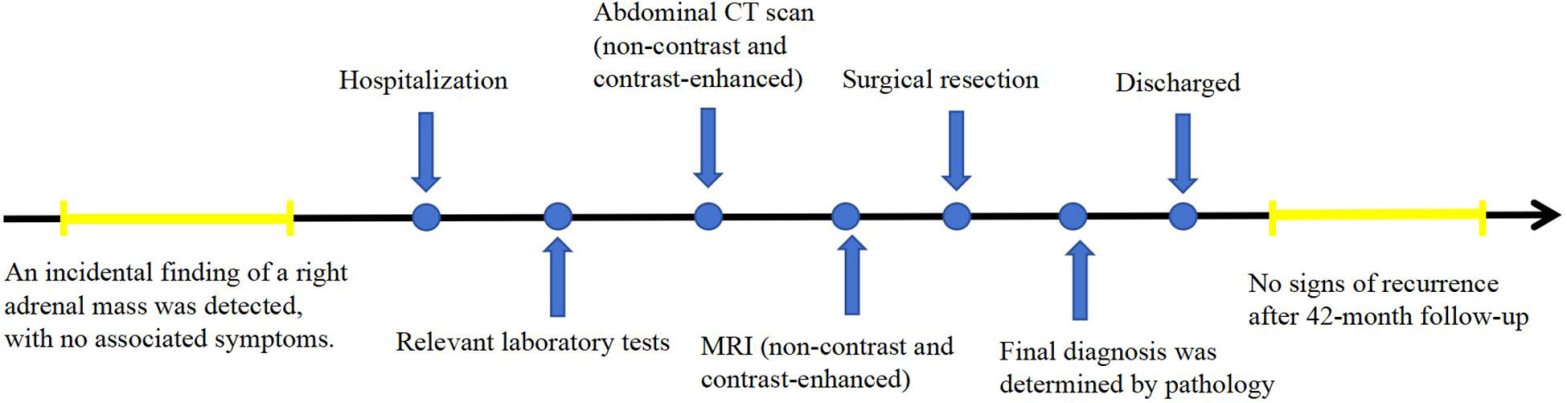
The timeline of diagnosis and treatment for case 1.

**Figure 2:**
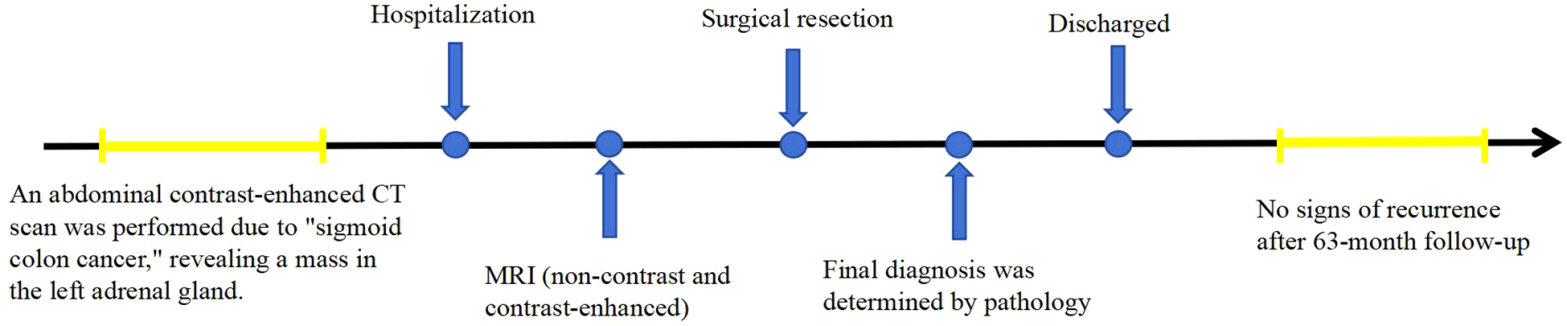
The timeline of diagnosis and treatment for case 2.

This report presents the CT and MR findings of two cases of adrenal hemangioma to raise awareness of this condition. In these two cases, the patients were treated with surgery at Jining No.1 People’s Hospital, Shandong Province. In the first case, the patient was followed up for 42 months postoperatively, and in the second case, the patient was followed up for 63 months postoperatively, with no signs of recurrence in either case. The timelines of diagnosis and treatment are shown in **Figures 1** and **2**.

## 2 Case description

### 2.1 Case 1

**Figure 3:**
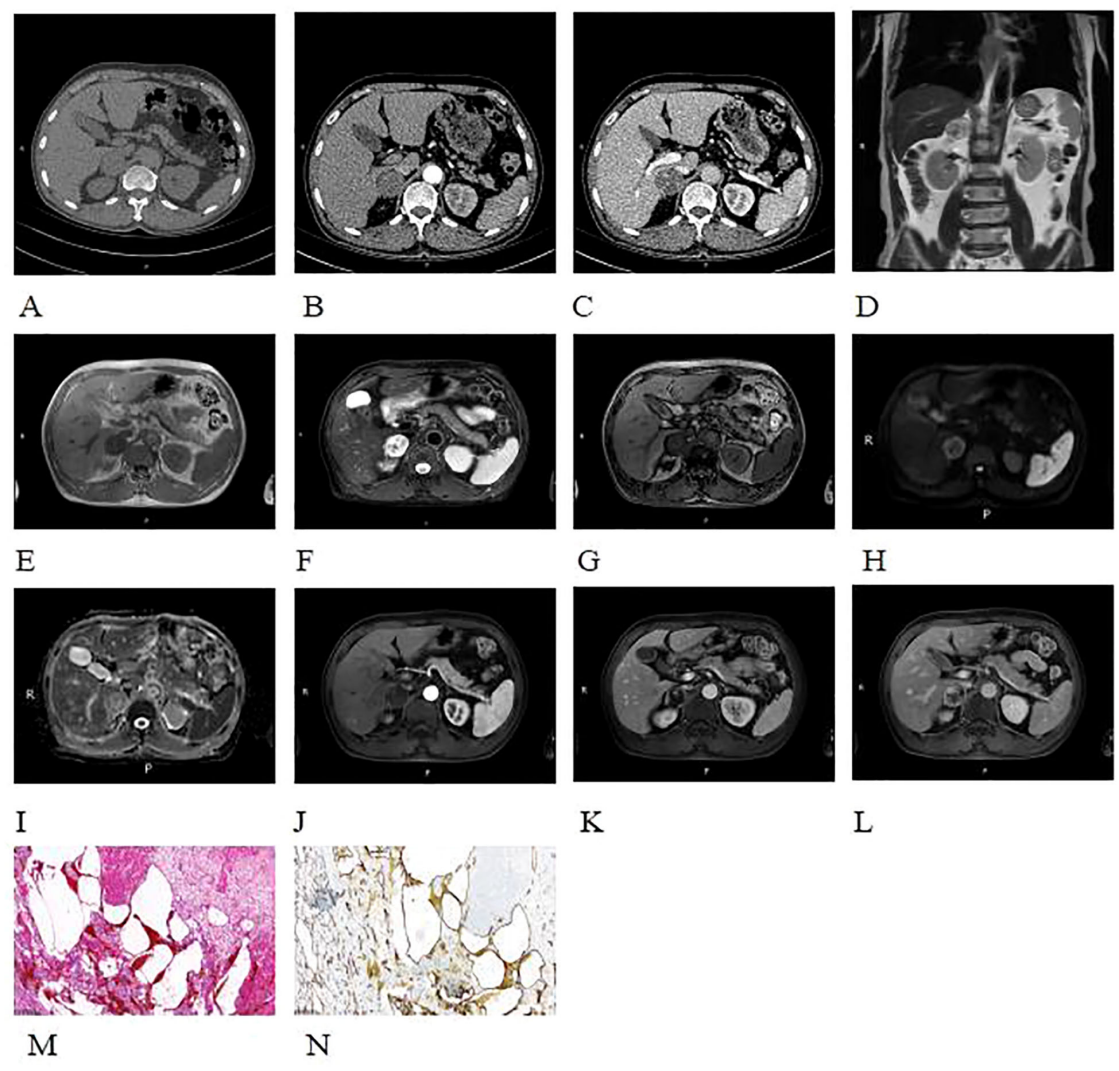
Plain CT images revealed a slightly hypodense, quasi-circular mass in the right adrenal gland **(A-C)**. On non-enhanced contrast MRI images, the lesion showed slightly hypointense signals on T1WI and mixed high and low signals on T2WI **(D, E)**. On T2WI-FS imaging, the lesion demonstrated a heterogeneous high and low signal intensity **(F)**. And no appreciable signal drop on the out-of-phase images **(E, G)**. DWI shows peripheral nodular-like high signal **(H)**, with no corresponding reduction in ADC map **(I)**. On contrast-enhanced CT and MR imaging, the lesion demonstrated nodular peripheral enhancement during the arterial phase, progressive centripetal filling in the venous phase, and incomplete filling with persistent enhancement expansion on delayed phases **(B, C, J-L)**. A well-defined capsular enhancement was observed **(L)**. Histopathological examination with H&E staining (×100) **(M)** revealed cystic spaces of varying sizes with hemorrhage within the adrenal gland, with a small amount of endothelial cells lining the cystic walls. Immunohistochemistry (×100) **(N)** showed CD34 positivity (+), indicating a vascular origin.

A 68-year-old male patient was incidentally found to have a right adrenal mass during a routine CT scan. The patient was asymptomatic, and relevant laboratory tests (such as cortisol, renin, angiotensin, and aldosterone) showed no abnormal findings. Plain CT images revealed a slightly hypodense, quasi-circular mass in the right adrenal gland measuring approximately 3.4 cm × 3.6 cm × 3.5 cm, with well-defined margins (**Figures 3A-C**). On MRI plain images, the lesion showed slightly hypointense signals on T1-weighted imaging (T1WI) and mixed high and low signals on T2-weighted imaging (T2WI) (**Figures 3D, E**). On T2WI-FS imaging, the lesion exhibited mixed high/low signal intensity (**Figure 3F**). Chemical shift imaging demonstrated no signal loss within the lesion on in-phase/opposed-phase sequences (**Figures 3E, G**). DWI revealed heterogeneous hyperintensity (**Figure 3H**), while the corresponding apparent diffusion coefficient (ADC) map showed no signal reduction (**Figure 3I**). On contrast-enhanced CT and MR imaging, the lesion demonstrated nodular peripheral enhancement during the arterial phase, progressive centripetal filling in the venous phase, and incomplete filling with persistent enhancement expansion on delayed phases (**Figures 3B, C, J-L**). Additionally, a well-defined capsular enhancement was observed (**Figure 3L**).

Following detailed discussion with the patient, laparoscopic right adrenalectomy was performed. At surgery, a mass measuring 3 cm × 2 cm, oval in shape, was observed in the right adrenal region, compressing the normal adrenal tissue into an elongated shape and adhering to surrounding structures.

Histopathological examination with H&E staining (**Figure 3M**) revealed cystic spaces of varying sizes showing hemorrhage within the adrenal gland and a small number of endothelial cells lining the cystic walls. Immunohistochemistry was performed to detect diagnostic markers. Among them, immunostains showed focal positivity for SMA, vascular positivity for CD34, negativity for CD117, DOG-1, S-100, and SOX-10, focal positivity for CK, sparse positivity for CgA, partial positivity for Inhibin-a, and a Ki-67 index of approximately 2%. These findings are consistent with a diagnosis of adrenal hemangioma.

### 2.2 Case 2

**Figure 4:**
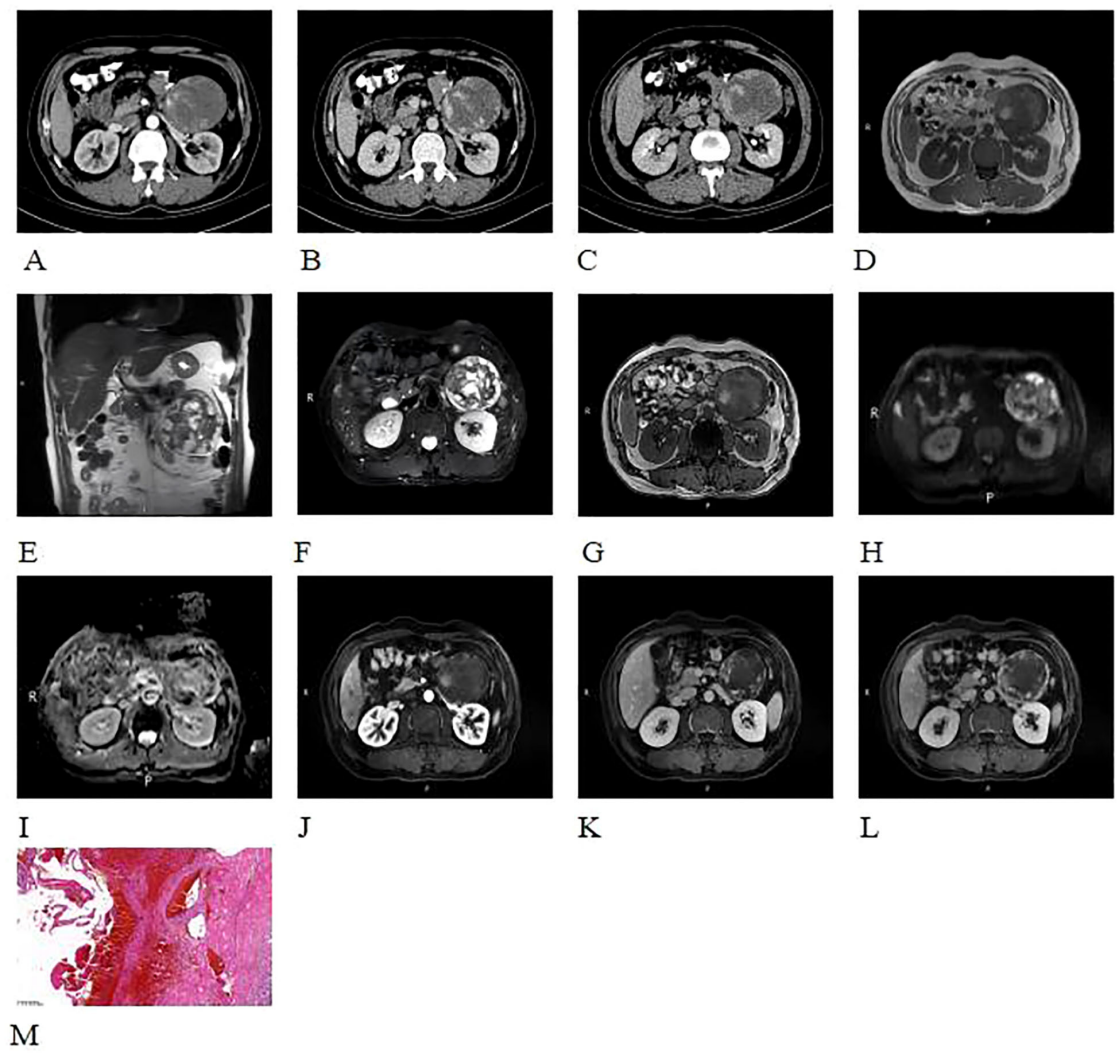
CT contrasted enhanced images revealed a quasi-circular mass with mixed density in the left adrenal gland, displaying indistinct margins and extending towards the tail of the pancreas **(A-C)**. The lesion demonstrated heterogeneous signal characteristics: low, isointense, and slightly hyperintense on T1WI **(D)**; isointense, slightly hyperintense, and hyperintense on T2WI **(E)**; mixed hyper- and hypointense on T2WI-FS **(F)**. No signal drop-off was observed on chemical shift imaging **(D, G)**. DWI revealed peripheral nodular hyperintensity **(H)** without corresponding ADC reduction **(I)**, and a discernible capsule was noted. Following contrast administration (CT/MRI), the lesion exhibited peripheral nodular and mass-like enhancement in the arterial phase **(A-C, J-L)**, progressed to centripetal filling in the venous phase, and showed expanded but incomplete enhancement with persistent non-enhancing areas in the delayed phase **(A-C, J-L)**, accompanied by a peripherally enhancing capsule **(L)**.Histopathological examination with H&E staining (×100) **(M)** showed abnormally proliferating blood vessels with extensive hemorrhage of the adrenal gland. The blood vessels varied in size and shape, with no atypia of the endothelial cells.

A 52-year-old male patient was found to have a left adrenal mass on abdominal CT with contrast enhancement, conducted due to “sigmoid colon cancer.” The patient did not undergo adrenal hormone tests. CT contrasted enhanced images revealed a left adrenal quasi-circular mass with mixed density lesion, measuring approximately 6.9 cm × 8.7 cm × 8.4 cm, with indistinct margins adjacent to the tail of the pancreas (**Figures 4A-C**). On unenhanced MRI, the lesion demonstrated heterogeneous signal characteristics: low, isointense, and slightly hyperintense on T1WI (**Figure 4D**); isointense, slightly hyperintense, and hyperintense on T2WI (**Figure 4E**); mixed hyper- and hypointense on T2WI-FS (**Figure 4F**). No signal drop-off was observed on chemical shift imaging (**Figures 4D, G**). DWI revealed peripheral nodular hyperintensity (**Figure 4H**) without corresponding ADC reduction (**Figure 4I**), and a discernible capsule was noted. Following contrast administration (CT/MRI), the lesion exhibited peripheral nodular and mass-like enhancement in the arterial phase (**Figures 4A-C, J-L**), progressed to centripetal filling in the venous phase, and showed expanded but incomplete enhancement with persistent non-enhancing areas in the delayed phase (**Figures 4A-C, J-L**), accompanied by a peripherally enhancing capsule (**Figure 4L**).

Following detailed discussion with the patient, exploratory laparotomy was performed. At surgery, a 10 cm × 8 cm × 8 cm tumor was found in the left retroperitoneum, which was mobile and had an indistinct margin adjacent to the left adrenal gland.

Histopathological examination with H&E staining (**Figure 4M**) showed abnormally proliferating blood vessels with extensive hemorrhage in the adrenal gland. The blood vessels varied in size and shape, without endothelial atypia, consistent with an adrenal hemangioma showing extensive hemorrhage and necrosis. No further immunohistochemical testing was performed.

## 3 Discussion

Adrenal hemangioma is a rare benign tumor originating from the adrenal stromal tissue, and its pathogenesis remains unclear (3). Histologically, adrenal hemangiomas are classified into four types: cavernous hemangioma, venous hemangioma, capillary hemangioma, and mixed-type hemangioma, with cavernous hemangioma being the most common (4). 

Adrenal hemangiomas typically occur in adults (3), and both of our cases involved elderly male patients. When the tumor is small, it typically presents no clinical symptoms, as seen in Case 1. However, when the tumor is larger and compresses surrounding structures, it may cause abdominal pain, as observed in Case 2 (2). Most adrenal hemangiomas do not secrete hormones; however, when associated with other adrenal disorders, they may produce corticosteroids (5). Duan et al. proposed (3)that adrenal hemangiomas are mostly unilateral (19/21), with the majority (18/22) having well-defined borders. In our cases, both tumors were unilateral, with one on the right side and the other on the left. One tumor was small with well-defined borders, while the other was larger, with an indistinct boundary adjacent to the tail of the pancreas.

The typical imaging features of adrenal hemangiomas are similar to those of hepatic hemangiomas. On plain CT, adrenal hemangiomas appear slightly hypodense (6). On T2WI-FS images, they demonstrate high signal intensity due to necrosis, hemorrhage, and scar formation, resulting in heterogeneous density and signal intensity. Calcification may also be present. Since they lack lipids, signal reduction is not observed on out-of-phase imaging. DWI shows heterogeneous high signal intensity, with no restriction of diffusion. On contrast-enhanced CT or MRI scans, the arterial phase shows significant peripheral nodular enhancement, while the venous and delayed phases exhibit centripetal filling (7), with a gradual decrease in enhancement. The capsule shows ring-like enhancement. The aforementioned imaging features are helpful in diagnosing this condition. The two cases we reported clearly exhibit the typical imaging characteristics of adrenal hemangioma. Duan et al. analyzed 21 cases of adrenal hemangioma and identified six patterns of enhancement (3): (1) peripheral nodular enhancement with delayed centripetal filling; (2) peripheral nodular enhancement without gradual centripetal filling; (3) nodular peripheral and central enhancement with progressive partial filling; (4) mild enhancement of the capsule and/or septa; (5) nodular or patchy central enhancement with progressive partial filling; (6) no enhancement. This classification of enhancement patterns enriches the imaging features of adrenal hemangioma and helps differentiate it from other adrenal lesions.

Adrenal hemangioma should be differentiated from other adrenal tumors (**Table 1**), including Adrenocortical carcinoma (ACC), Pheochromocytomas, Adrenal adenomas and Anastomosing hemangioma, due to their differing imaging features and clinical implications.

**Table 1:** The radiological feature differentiation between adrenal hemangioma, adrenal adenoma, adrenocortical carcinoma, and pheochromocytomas.

Differential characteristics	Plain CT	T2WI(MRI)	Enhancement characteristics	Chemical Shift Imaging (out-of-phase)	DWI	^131^I-MIBG imaging
Adrenal hemangioma	Equal/low density with or without calcification	Significant high signal	Peripheral nodular-like gradual centripetal filling	No signal loss	No restriction	No uptake
Adrenal adenomas	<10 HU(Fat-rich type)	Equal/slightly high signal	Uniform enhancement with rapid peripheral clearance	Significant signal loss	No/mild restriction	No uptake
Pheochromocytomas	Heterogeneous density	Significant high signal	Significant enhancement with slow peripheral clearance	No signal loss	Some are restricted.	Significant focal uptake
Adrenocortical carcinoma	Mixed density with calcification/necrosis	Moderate/high heterogeneous signal	Irrergular, persistent enhancement	No signal loss	Significant restriction	Most show no uptake (occasionally weak uptake observed)

ACC masses are typically large and irregular in shape. In 30% of cases, small or coarse calcifications can be observed, often located at the center of the tumor. The solid component of the tumor shows restricted diffusion on DWI. On contrast-enhanced CT or MRI scans, the solid part of the tumor rapidly enhances during the arterial phase and continues to enhance in the delayed phase (8). ACC typically demonstrate no radiotracer uptake on Meta-^[131^I]iodobenzylguanidine (^131^I-MIBG) scintigraphy (9). However, a small proportion of ACC may exhibit uptake on somatostatin analogs (SAs) (10).

Pheochromocytomas commonly occur in the adrenal medulla and often present with typical clinical manifestations (11). These include sustained or paroxysmal hypertension, headache, palpitations, and sweating, all caused by hormone excess. However, the clinical presentationis highly variable and can mimic many other diseases. Due to associated hemorrhage, necrosis, and cystic changes, the imaging often shows heterogeneous density and signal intensity. On contrast-enhanced scans, the arterial phase shows significant enhancement, without a tendency for progressive filling (12). Radiolabeled SAs in adrenal imaging show significant uptake of SAs in Pheochromocytomas. ^131^I-MIBG imaging can effectively distinguish Pheochromocytomas from other types of adrenal tumors, since Pheochromocytomas showing specific uptake (9, 10).

Adrenal adenomas are more commonly unilateral and, due to their high lipid content, may show signal reduction on out-of-phase imaging. On contrast-enhanced scans, the mass rapidly enhances and promptly clears (13). Adrenal adenomas exhibit relatively low uptake of SAs. However, in ^131^I nor-cholesterol adrenal scintigraphy, adrenal adenomas show radioactive uptake, making it an effective tool for distinguishing benign adrenal tumors (9, 10).

Anastomosing hemangioma is a rare benign vascular tumor that occurs in the kidneys, perirenal fat, liver, ovaries, small intestine, and adrenal glands, with adrenal gland involvement accounting for 3.2%. On CT scan, the tumor exhibits well-defined margins and hyperdensity relative to background parenchyma, with persistent enhancement in the venous and delayed phases. Correspondingly, on MRI scan, it typically demonstrates hypointense signal on T1WI and hyperintense signal on T2WI. The imaging features of anastomosing hemangioma in the adrenal gland overlap with those of adrenal hemangioma, and the diagnosis primarily relies on histopathological examination (14).

Although optimal treatment guidelines for adrenal hemangiomas have not been established, surgical intervention is recommended when the tumor exceeds 6 cm due to its tendency for bleeding and the inability to exclude malignant components. For tumors between 4 and 6 cm, the patient’s preferences and clinical condition should be considered. For non-functional tumors under 4 cm, a conservative strategy of active monitoring may be appropriate, guided by periodic endocrinological and radiological evaluations (6, 15, 16).

## 4 Conclusion

Adrenal hemangioma is a rare benign tumor originating from the adrenal stromal tissue. Typical adrenal hemangiomas show high signal intensity on T2WI-FS images. Signal intensity appears heterogeneous when hemorrhage, necrosis, or scar formation is present. Dynamic studies demonstrate peripheral nodular and centripetal enhancement, with the capsule exhibiting ring-like enhancement. Atypical adrenal hemangiomas should be distinguished from adrenal adenomas, adrenocortical carcinoma, and Pheochromocytomas. Surgical intervention is recommended for adrenal hemangiomas larger than 6 cm. For tumors between 4 and 6 cm, the patient’s preferences and clinical condition should be considered. Follow-up observation may be considered for patients with non-functional tumors smaller than 4 cm.

## Data Availability

The original contributions presented in the study are included in the article/supplementary material. Further inquiries can be directed to the corresponding author.
